# Cross-Sectional Association between Behaviors Related to Sugar-Containing Foods and Dental Outcomes among Hispanic Immigrants

**DOI:** 10.3390/ijerph17145095

**Published:** 2020-07-15

**Authors:** Sonia Vega-López, Karla Armenta, George Eckert, Gerardo Maupomé

**Affiliations:** 1 College of Health Solutions, Arizona State University, Phoenix, AZ 85004, USA; 2 Southwest Interdisciplinary Research Center, Arizona State University, Phoenix, AZ 85004, USA; 3 SDSU Health LINK Center for Transdisciplinary Health Disparities Research, San Diego State University Research Foundation, San Diego, CA 92120, USA; klarmenta@sdsu.edu; 4 School of Medicine, Indiana University/Purdue University in Indianapolis, Indianapolis, IN 46202, USA; geckert@iu.edu; 5 Richard M. Fairbanks School of Public Health, Indiana University/Purdue University in Indianapolis, Indianapolis, IN 46202, USA; gmaupome@iu.edu; 6 Indiana University Network Science Institute, Indiana University, Bloomington, IN 47408, USA

**Keywords:** sugar-containing foods, Hispanic, immigrants, dental outcomes

## Abstract

A cross-sectional, self-administered survey was used to gather information about dental outcomes, sugar-containing food behaviors and intake, and sociodemographic characteristics of adults of Mexican and Central-American (Guatemala, El Salvador, Honduras) origin (*n* = 517). Bivariate and multiple-variable logistic regressions were used to examine the associations of behaviors related to added sugar-containing foods/beverages (overall intake and consumption before bed) with dental outcomes. Outcome measures involved dental outcomes, dental self-care practices, and added sugar intake. Estimated daily added sugar intake among all participants was 98 (SD = 99) g, with no difference in consumption among participants from different countries. The majority of added sugar (63 (SD = 74) g) was provided by sugar-sweetened beverages. Participants who reported consuming sugar-containing foods or beverages within an hour before bed were more likely to report having a fair/poor/very poor condition of teeth and gums and having felt dental pain (*p* < 0.05 for all). The amount of sugar intake was associated with being prescribed medication for oral or dental problems (*p* = 0.008) and dental pain (*p* = 0.003). Findings support the association between sugar containing food–related behaviors and dental problems among Hispanic immigrants to the U.S. Health promotion and preventive interventions for this population should consider these behaviors as modifiable contributors to adverse dental outcomes.

## 1. Introduction

Despite the efforts directed at eliminating health disparities, Hispanics, the largest minority group in the U.S. [1], are at higher risk for developing chronic diseases relative to other ethnic groups [2,3] in part because of their lack of adherence to dietary recommendations [4,5]. Moreover, Hispanics continue to have one of the highest prevalence rates of caries and unmet oral treatment needs [6,7].

Studies assessing the diet of Hispanics in the U.S. support a need for dietary improvement; with high consumption of sugar-containing foods and beverages being a concern [8,9]. In a report on 1000 U.S. Hispanics, 58% reported consuming sugar-sweetened beverages more than twice daily [10]. Although National Health and Nutrition Examination Survey (NHANES) data suggest that overall sugar-sweetened beverage consumption in the U.S. is declining, consumption among Hispanics continues to surpass recommended levels, particularly among individuals with a lower income [8].

Among Hispanics, intake of sugar-containing foods and beverages has been associated with greater rates of obesity, insulin resistance, and diabetes [11,12]. Consumption of sugar-containing foods and beverages has also been associated with dental caries [13,14,15]. While high-sugar intake has been associated with negative dental outcomes among some Hispanics [16], this large ethnic group in the U.S. encompasses highly diverse groups; which include multiple nationalities of origin, how recently they relocated to the U.S., degree of adoption of mainstream customs for food and dental care, and their varying ability to navigate health care systems. Despite having some commonalities, the risk factors and diets of Hispanics from different countries also have unique traits ascribable to their region of residence and country of origin [4,17].

The association between higher intake of sugar-containing foods and beverages and negative dental outcomes appears to follow well-established trends for non-Hispanics and almost every racial/ethnic group. Our own research in 326 immigrant and U.S.-born adults of Mexican origin showed that those with greater added sugar intake were more likely to report having had a toothache, and having been prescribed antibiotics and analgesics for dental reasons in the preceding 12 months [16]. Having food or drinks within one hour of bedtime did emphasize some effects, as summarized in a recent systematic review [18]. Similarly, in a sample of 308 older adults, 40% of whom were Hispanics, tooth loss was positively associated with the consumption of one, or more than one, sugar-sweetened beverage per day [19].

Direct evidence is lacking regarding potential differences in diet and risk factors for dental problems across Hispanics with diverse nationalities of origin, as well as the expanding migration trends of individuals from Central America in recent times. Therefore, the aim of the present study was to assess the relationship of behaviors related to sugar-containing foods (overall intake and consumption within an hour of bed) with dental outcomes among an understudied population: immigrants from Mexico, Guatemala, El Salvador, and Honduras to the U.S. In contrast to previous work, the present study assessed sugar intake using an instrument that incorporated culturally specific beverages, foods, and snacks relevant to the target population.

## 2. Materials and Methods

This cross-sectional analysis was conducted using data from the TalaSurvey Study [20,21] and the DentCHeP Study. All study procedures were approved by the Indiana University Institutional Review Board (#1306011692 and #1709401236), and were offered in English or Spanish, according to participant preference.

### 2.1. Study Participants

Participants were urban-based adults, first- or second-generation immigrants of Mexican and Central-American (Guatemala, El Salvador, Honduras) origin, who could read and write English or Spanish. There were no additional inclusion/exclusion criteria for participation. For the sake of simplicity, participants are referred in aggregate as “Hispanics”. They were recruited in 2013 and 2017 through flyers, posters, and public announcements made in community-based organizations, parishes, and businesses and agencies serving the Hispanic community in greater Indianapolis, IN. Interested participants received a thorough description of study procedures after which they had the opportunity to have their questions answered by bilingual staff. All participants provided written informed consent prior to completing a self-administered survey, available in English and Spanish at a third-grade reading level. The survey asked questions about sociodemographic characteristics, dental outcomes and self-care, and sugar-containing food behaviors and intake. Participants had access to bilingual staff while they were completing the survey if they needed clarification of any questions or answer options.

### 2.2. Survey Measures

Participants self-reported their age (in years), education (none, some elementary school, elementary school complete, some middle school, middle school complete, high school complete, university degree), marital status (single, married or in domestic partnership, separated/divorced, widowed), medical and dental insurance coverage (yes/no), and U.S. country of origin.

Questions regarding dental outcomes and dental self-care practices were assembled from the National Institute of Dental and Craniofacial Research (NIDCR)/Centers for Disease Control and Prevention (CDC) Data Resource Center collection of national surveys [22] and included: How would you describe the condition of your teeth and gums? (Yes = excellent/very good/good; No = fair/poor/very poor/no natural teeth), During the past 12 months, did you have pain in your teeth (a dull, aching pain across your face or cheek)? (Yes/No), During the past 6 months, how often did you suffer from sore or bleeding gums? (Yes = often/sometimes; No = rarely/never), Have you been prescribed antibiotics or painkillers because of problems with your teeth in the past year? (Yes/No), During the past 6 months, did you take over-the-counter medicine (e.g., Tylenol, Advil, ibuprofen, aspirin) for problems with your mouth, gums, or teeth? (Yes/No), How many of your adult teeth have been removed specifically because of tooth decay or gum disease? (Yes = answer ≥1 tooth), During the past 6 months, how often were you self-conscious or embarrassed because of the way your mouth looks? (often/sometimes/rarely/never), During the past 6 months, how often were you self-conscious or embarrassed because of the way your mouth smells? (often/sometimes/rarely/never), During the past 6 months, how often did you feel that life in general was less satisfying because of problems with your mouth or your breath? (very often/occasionally/hardly ever/never), During the past 6 months, how often did you avoid particular foods because of problems with your mouth? (very often/occasionally/hardly ever/never).

To estimate added sugar intake, participants completed the Added Sugar Intake Estimate survey (ASIE) [16]. The ASIE was developed after extensive formative research with key stakeholders from organizations serving the Hispanic population in Indiana, with the purpose of culturally tailoring questions from the National Cancer Institute Diet History Questionnaire II (NCI-DHQII) [23] in order to include representative sugar-containing foods, snacks, and beverages commonly consumed by individuals of Mexican origin. For the present report, additional formative research, consisting of key informant interviews and focus groups and overseen by an advisory board of Hispanic community members, was conducted to adapt the survey tool to Honduran, Guatemalan, and Salvadorian idioms; terminology; and relevant foods, snacks, and drinks with high added sugar content. The resulting 22-item ASIE includes the following items: milk-based frozen desserts; puddings and custards; chocolate; non-chocolate candy; syrup, fruit preserves, and sweet spreads; cake and pastries; cookies; pies and fruit-containing pastries; sherbets and water-based frozen desserts; water; plain milk; powdered, evaporated, and condensed milk; flavored milk; regular soda; non-fruit sweetened beverages (e.g., Kool-Aid); sports drinks; energy drinks; 100% fruit and/or vegetable juices; fruit-flavored drinks; sweetened coffee; and sweetened teas. The survey follows a semi-quantitative food frequency format in which frequency of consumption for each category of foods or beverages is asked first, with response options of never, a few times per year, once a month, 2–3 times a month, once a week, twice a week, 3–4 times a week, almost every day, and every day. Participants are then asked to report the portion size and the number of portions consumed on average, using visuals of actual standard-portion plates and cups to help participants estimate portion size. In order to calculate daily sugar intake, annualized frequency of intake reported by participants is then multiplied by the number of portions and portion size reported, and by the amount of sugar in a standard portion of foods in each category reported in the United States Department of Agriculture (USDA) National Nutrient Database [24], and then divided by 365.25. The resulting value is reported as estimated daily sugar intake (g/day).

Each food/beverage item in the ASIE is followed by the question: I often eat (food/beverage) within one hour of going to bed (Yes/No). Answers to whether respondents drank/ate sugar-containing foods or beverages within one hour of going to bed were summed into three separate variables and coded as “Yes” if participants responded affirmatively to at least one food (eat snacks/foods within 1 h before bedtime), beverage (drink within 1 h before bedtime), or sugar-containing item (eat or drink anything within 1 h before bedtime).

### 2.3. Statistical Analysis

Sociodemographic and dental questionnaire items and daily added sugar intake were summarized overall and by country of origin. The natural logarithm of daily added sugar intake was used for the analyses to achieve normality. One-way ANOVA was used to compare country of origin for differences in daily added sugar intake; post hoc, pair-wise tests were performed when the overall test was significant. Associations of food and/or drink consumption before bed and daily sugar intake with dental outcomes were individually assessed using chi-square tests and logistic regression, respectively. Multiple-variable logistic regression was then used to simultaneously examine the effects of food and/or drink consumption before bed and daily sugar intake on dental outcomes while accounting for sociodemographic characteristics. Education level (treated as a continuous variable), marital status, health insurance, and dental insurance were included as covariates in all models, regardless of statistical significance. Three variables for sugar-containing foods or drinks before bed were considered (food, beverage, and either food or beverage), and the most significant of the three variables was retained in the models. Three variables for sugar intake were also considered (total, food, beverage), and again the most significant of the three variables was retained in the models. Regression/beta coefficients and odds ratios are presented for each predictor. The area under the Receiver Operating Characteristic (ROC) curve for the model was calculated to represent how well the model distinguishes between the two levels of each dental outcome. All analyses were undertaken in SAS 9.4 (SAS Institute Inc., Cary, NC, USA). A 5% significance level was used for all tests.

## 3. Results

Participants who completed the survey (*n* = 517) included 332 from Mexico, 53 from Guatemala, 63 from El Salvador, and 69 from Honduras. Six participants of Mexican origin were excluded from the analysis: one reported implausibly high added sugar intake (>1250 g/day) and the other five had incomplete responses for five or more food categories. The final analyses included 326 Mexican and 185 Central-American participants. A majority of participants were married or living with a partner (63%); 32% reported being single; and 5% reported being separated, divorced, or widowed. Participants’ reported level of education was lower than middle school for about a third of participants (33%); 27% reported completing middle school, 18% reported completing high school, and 10% reported having a college degree. Most participants (70%) reported only speaking Spanish. More than half of participants reported having medical or dental insurance (52% and 59%, respectively).

Participants’ self-reported dental outcomes and dental self-care practices are listed in Table 1. Only about a third of all participants considered their teeth and gums to be in excellent, very good, or good condition, with fewer Guatemalans (25%) and Salvadorians (19%) giving this qualification to the condition of their teeth. About a third of participants reported having had pain in their teeth over the past 12 months or having had bleeding gums over the past 6 months. About 15% of participants reported having been prescribed antibiotics or painkillers, and about 30% reported using over-the-counter medications due to dental issues in the past 12 months. About half of all participants reported having had at least one tooth removed due to decay, with this proportion being greater (60%) among Guatemalans. About 40% of participants reported feeling embarrassed often or sometimes because of how their mouth looks or smells. About a third of participants reported feeling that life is less satisfying very often (4%) or occasionally (26%) because of oral health problems. About a third of participants also reported avoiding specific foods very often (4%) or occasionally (26%) due to oral health issues.

About two-thirds of participants (68%) reported often consuming at least one type of sugar-containing food or beverage within 1 h of bedtime; 49% of participants reported consuming sugar-containing food/snacks and 58% reported consuming sugar-containing beverages before bedtime. Table 2 depicts the comparison of estimated daily added sugar intake based on participants’ country of origin. Estimated daily added sugar intake among all participants was 98 (SD = 99; median = 66) g, with Salvadorians consuming the least amount (69 (SD = 60; median = 59) g; n.s.). The majority of estimated added sugar (63 (SD = 74; median = 41) g) was obtained through consumption of sugar-sweetened beverages. Given the lack of statistical differences in estimated added sugar intake by country of origin, all subsequent analyses were conducted grouping all participants together.

Results from bivariate analyses exploring associations of dental outcomes based on behaviors related to sugar-containing foods are displayed in Table 3. Participants who consumed sugar-containing foods within an hour before bed were more likely to report having a fair/poor/very poor condition of teeth and gums (*p* = 0.002) and having felt dental pain (*p* = 0.028).

Similarly, participants who consumed sugar-containing beverages within an hour before bed were more likely to report having a fair/poor/very poor condition of teeth and gums (*p* = 0.003), avoiding foods because of problems with their mouth more often (*p* = 0.012), considering life less satisfying because of problems with their mouth/breath more often (*p* = 0.046), having had sore or bleeding gums (*p* = 0.026), and having had dental pain (*p* = 0.026). Greater added sugar intake from food was associated with participants reporting having had medications prescribed for oral or dental problems (*p* = 0.003) and having had dental pain (*p* = 0.018). Greater added sugar intake from beverages was associated with participants reporting having had sore or bleeding gums more frequently (*p* = 0.036), having had medications prescribed for oral or dental problems (*p* = 0.004), and having had dental pain (0.002). Additional analyses indicated no statistical association between diet-related behaviors and being embarrassed because of mouth smell, being embarrassed because of mouth appearance, use of over-the-counter medicine for oral or dental problems, or having had teeth removed (data not shown).

In multivariate analyses (Table 4), consuming sugar-containing food and/or drink before bed was associated with reporting a worse condition of teeth and gums (*p* = 0.001) and sore or bleeding gums (0.019); consuming food before bed was associated with being less embarrassed because of mouth smell (*p* = 0.025). The amount of sugar intake was associated with being prescribed medication for oral or dental problems (*p* = 0.008) and dental pain (*p* = 0.003). No significant associations were found with consuming sugar-containing foods or drinks before bed or added sugar intake in models that included being embarrassed because of mouth appearance, avoiding foods because of problems with the mouth, life being less satisfying because of mouth or breath problems, taking over-the-counter medicine for oral or dental problems, or having had teeth removed (data not shown).

## 4. Discussion

Hispanics have one of the highest reported prevalence rates of dental problems [6,7] as well as elevated reported consumption of sugar-containing foods and beverages [8,9,10,25]. Although the relationship between sugar intake and negative dental outcomes is already established [13,16], information about how sugar intake-related behaviors influence negative dental outcomes among Hispanics is limited. In the current study, a large proportion of participants reported perceiving having their teeth and gums in fair or poor condition, having dental health issues (e.g., tooth loss, use of medications for dental pain), and having dental issues that affect different aspects of life (e.g., less life satisfaction, embarrassment because of their teeth appearance). This is not surprising given the study recruited participants from a vulnerable population (immigrants from Mexico, Guatemala, El Salvador, and Honduras), with overall low levels of education and income, and potentially low access to the medical system due to low medical and dental insurance rates. Overall findings suggest that participants who reported consuming sugar-containing foods or beverages within an hour before bed were more likely to report having a fair/poor/very poor condition of teeth and gums and having felt dental pain. The amount of sugar intake was associated with being prescribed medication for oral or dental problems and dental pain.

Similar to what was observed in prior work [16], current study participants had a high estimated added sugar intake, ranging between 69 g for Salvadorians and 111 g for Guatemalans (n.s.). In 2005–2010 national surveillance data, Mexican-Americans reported 13% of energy intake from added sugars [26]. Among all U.S. adults in 2011–2012, added sugars provided about 17% of their total energy intake [27]. Existing evidence supports that consuming <10% of energy intake from sugars is associated with lower dental caries [15]. In the context of a 2000 kcal diet, this would represent consuming no more than 50 g of total sugars, including those naturally occurring in fruit, vegetables, and dairy products. The high added sugar intake reported in the current study, in addition to greater sugar intake being associated with dental pain and the use of medications for dental problems, points to an urgent need for reducing sugars intake in this population.

Consumption of added sugars from sugar-sweetened beverages was of interest given the well-documented contribution of sugar-sweetened beverages to the development of adverse dental outcomes [13], in addition to the fact that the majority of added sugar consumed by study participants was obtained through sugar-sweetened beverages. Nationally representative data suggest that adults of Hispanic descent, as well as those with lower education and lower and middle income are more likely to report consuming sugar-sweetened beverages than their non-Hispanic white, high-education, and high-income counterparts, respectively [8]. Interestingly, the greater consumption of sugar-sweetened beverages for Hispanics was associated with a greater likelihood to consume fruit drinks, rather than soda or energy/sports drinks intake [8]. This may be related to the perception of fruit drinks (natural or fruit-flavored) as a healthful component of the diet [28,29].

Consumption of sugar-containing foods is a known risk factor for developing caries [30]. It is noteworthy that two-thirds of current study participants reported often consuming sugar-containing foods or beverages within 1 h of bedtime, which was associated with reporting a worse condition of their teeth and gums, and in the case of sugar-sweetened beverages, with more dental pain. Prior work conducted with adults of Mexican origin suggested that individuals who eat or drink sugar-containing foods before bedtime are more likely to consume greater amounts of added sugars [16].

The present study offers limited support to confirm that these four nationalities of origin (Mexico, Guatemala, El Salvador, and Honduras) do not appear to differ for added sugar consumption in important ways; considering that they make up about 80% of the Hispanic population in the U.S. and in Indiana, the lack of major differences offers a measure of certainty about using similar approaches to address the large intake of added sugars across people of Mexican, Honduran, Salvadorian, and Guatemalan origins. Despite shedding light on added sugar intake and its relationship to key dental outcomes for such sparsely studied population groups, some limitations of the current study deserve attention. This study focused on a specific population of immigrants from Mexico, Guatemala, El Salvador, and Honduras to an expanding immigration gateway in the Midwestern U.S., limiting the generalizability of findings. Nevertheless, the study addresses a need to support this underserved and highly vulnerable population at great risk for dental and other medical problems. Due to the cross-sectional nature of this study, findings only point to associations between sugar-related behaviors and dental outcomes, and no causality can be established. Social desirability and selection biases associated with self-reported data and sampling methodologies cannot be ruled out; however, there are no obvious reasons to believe that study participants had reasons for disguising or attenuating sugar consumption reports. As with all self-reported data, objective verification in future research would be desirable. Moreover, because data were self-reported, it was unfeasible to accurately tease out other conditions contributing to pain experience, such as tooth fracture or abrasion. Finally, the dietary instrument used to assess sugar intake only accounted for added sugars and not those naturally occurring in foods [16]; we were unable to account for the mechanics of added sugar intake in the context of health- or lifestyle-related factors.

## 5. Conclusions

Findings from this study provide further evidence for the need to promote reductions in sugar intake among the highly vulnerable population of Hispanic migrants to urban Indiana based on associations between sugar intake and consuming sugar-containing food and beverages before bed with dental problems. Whereas the elevated sugar intake reported by study participants is not surprising given the wide availability, ease of access, and low cost of added sugar-containing foods and beverages, findings point to an urgent need for multi-level approaches to improve dietary quality in this population. From a behavioral standpoint, preventive interventions should focus on intake patterns (i.e., when sugar-containing foods and beverages are consumed) in addition to actual consumption amounts. From a health promotion perspective, accurate information about dietary changes over time and along acculturation continua appears to be a promising area for future research and program development. Finally, these patterns of added sugar intake emphasize the need to not only inform these vulnerable populations about health problems linked to risk factors but also to inform policy makers of the wider dimension of social determinants of health. In this larger dimension, food insecurity, food policy, and food prices and availability are major drivers for food choices.

## Figures and Tables

**Table 1:** Description of self-reported oral health-related outcomes and self-care practices *.

Outcome/Behavior	Mexico *n* (%)	Guatemala *n* (%)	El Salvador *n* (%)	Honduras *n* (%)	All *n* (%)
How would you describe the condition of your teeth and gums? YES if answer = excellent/very good/good (Condition of teeth/gums excellent/very good/good)					
No	201 (63%)	40 (76%)	51 (82%)	38 (56%)	330 (65%)
Yes	118 (37%)	13 (25%)	12 (19%)	30 (42%)	173 (34%)
During the past 12 months, did you have pain in your teeth (a dull, aching pain across your face or cheek)? (Toothache in the last 12 months)					
Yes	115 (35%)	23 (43%)	9 (15%)	22 (33%)	169 (33%)
No	211 (65%)	30 (57%)	53 (85%)	44 (67%)	338 (67%)
During the past 6 months, how often did you suffer from sore or bleeding gums? YES if answer = often/sometimes (Sore/bleeding gums in the last 6 months)					
Yes	115 (37%)	24 (45%)	16 (26%)	21 (31%)	176 (35%)
No	198 (63%)	29 (55%)	46 (74%)	48 (70%)	321 (65%)
Have you been prescribed antibiotics or painkillers because of problems with your teeth in the past year? (Antibiotics in the last 12 months)					
No	292 (90%)	35 (66%)	52 (83%)	54 (78%)	433 (85%)
Yes	34 (10%)	18 (34%)	11 (17%)	15 (22%)	78 (15%)
During the 6 months, did you take over-the-counter medicine (e.g., Tylenol, Advil, ibuprofen, aspirin) for problems with your mouth, gums, or teeth?					
No	262 (80%)	22 (42%)	36 (60%)	38 (55%)	358 (71%)
Yes	64 (20%)	30 (58%)	24 (40%)	31 (45%)	149 (29%)
How many of your adult teeth have been removed SPECIFICALLY because of tooth decay or gum disease? YES if answer ≥1 tooth (Any teeth removed due to caries/gum disease)					
No	174 (52%)	20 (40%)	30 (51%)	31 (47%)	255 (50%)
Yes	158 (48%)	30 (60%)	29 (49%)	35 (53%)	252 (50%)
During the past 6 months, how often were you self-conscious or embarrassed because of the way your mouth **looks**? (Embarrassed by the way mouth looks)					
Often	28 (10%)	7 (13%)	8 (13%)	3 (4%)	46 (10%)
Sometimes	102 (35%)	15 (28%)	11 (18%)	13 (19%)	141 (30%)
Rarely	74 (25%)	12 (23%)	10 (17%)	16 (23%)	112 (24%)
Never	88 (30%)	19 (36%)	31 (52%)	37 (54%)	175 (37%)
During the past 6 months, how often were you self-conscious or embarrassed because of the way your mouth **smells**? (Embarrassed by the way mouth smells)					
Often	12 (4%)	3 (6%)	2 (3%)		17 (4%)
Sometimes	110 (38%)	19 (36%)	15 (25%)	18 (26%)	162 (34%)
Rarely	85 (29%)	13 (25%)	21 (35%)	21 (30%)	140 (30%)
Never	83 (29%)	18 (34%)	22 (37%)	30 (43%)	153 (32%)
During the past 6 months, how often did you feel that life in general was less satisfying because of problems with your mouth or your breath? (Life less satisfying due to mouth/breath)					
Very often	14 (5%)	1 (2%)	3 (5%)	1 (1%)	19 (4%)
Occasionally	87 (29%)	14 (26%)	12 (19%)	14 (20%)	127 (26%)
Hardly ever	66 (22%)	9 (17%)	11 (17%)	11 (16%)	97 (20%)
Never	134 (45%)	29 (55%)	37 (59%)	43 (62%)	243 (50%)
During the past 6 months, how often did you avoid particular foods because of problems with your mouth? (Avoid foods because of mouth problems)					
Very often	14 (5%)	1 (2%)	3 (5%)	3 (4%)	21 (4%)
Occasionally	91 (29%)	13 (25%)	10 (16%)	12 (17%)	126 (26%)
Hardly ever	77 (25%)	11 (21%)	11 (18%)	9 (13%)	108 (22%)
Never	129 (41%)	27 (52%)	38 (61%)	45 (65%)	239 (48%)
I often eat (anything) within one hour of going to bed (Eat or drink within 1 h before bedtime)					
No	89 (27%)	19 (36%)	31 (49%)	24 (35%)	163 (32%)
Yes	237 (73%)	34 (64%)	32 (51%)	45 (65%)	348 (68%)
I often eat (food or snack) within one hour of going to bed (Eat snack/foods within 1 h before bedtime)					
No	154 (47%)	26 (49%)	40 (63%)	39 (57%)	259 (51%)
Yes	172 (53%)	27 (51%)	23 (37%)	30 (43%)	252 (49%)
I often drink (beverage) within one hour of going to bed (Drink within 1 h before bedtime)					
No	115 (35%)	30 (57%)	39 (62%)	33 (48%)	217 (42%)
Yes	211 (65%)	23 (43%)	24 (38%)	36 (52%)	294 (58%)

* Specific survey items are included with variable names noted in parenthesis. For dichotomized variables, answer options considered for “YES” are noted.

**Table 2:** Comparison of daily sugar intake (in g) by country of origin.

	Mexico (*n* = 326)	Guatemala (*n* = 53)	El Salvador (*n* = 63)	Honduras (*n* = 69)	All (*n* = 511)
Total	69 (0–544)	60 (6–776)	59 (1–273)	62 (0–414)	66 (0–776)
Food	20 (0–261)	16 (2–162)	12 (1–146)	18 (0–350)	19 (0–350)
Drink	43 (0–480) ^a^	36 (2–662) ^ab^	29 (0–170) ^b^	34 (0–405) ^ab^	41 (0–662)

Data shown as Median (Range). Values in the same row with different superscripts are significantly different (*p* < 0.05) based on a comparison of natural log-transformed data.

**Table 3:** Bivariate analyses predicting dental outcomes based on diet-related behaviors.

Diet-Related Behavior		Response				*p*-Value
		*n*	% or Mean (SD)	*n*	% or Mean (SD)	
Condition of teeth and gums						
		Very good/Good		Fair/Poor/Very Poor		
Food before bed	No	105	41%	152	59%	**0.002**
	Yes	68	28%	178	72%	
Drink before bed	No	89	42%	125	58%	**0.003**
	Yes	84	29%	205	71%	
Food/drink before bed	No	73	45%	89	55%	**0.001**
	Yes	100	29%	241	71%	
ln (Daily sugar intake)		156	4.1 (1.0)	302	4.1 (1.1)	0.532
ln (Daily sugar from food)		156	3.0 (1.1)	302	3.0 (1.2)	0.882
ln (Daily sugar from beverages)		156	3.5 (1.1)	302	3.6 (1.3)	0.409
Avoid foods because of problems with mouth						
		Often/Sometimes		Rarely/Never		
Food before bed	No	68	27%	187	73%	0.121
	Yes	79	33%	160	67%	
Drink before bed	No	51	24%	163	76%	**0.012**
	Yes	96	34%	184	66%	
Food/drink before bed	No	41	25%	120	75%	0.147
	Yes	106	32%	227	68%	
ln (Daily sugar intake)		139	4.0 (1.2)	310	4.1 (1.0)	0.557
ln (Daily sugar from food)		139	3.0 (1.2)	310	3.0 (1.1)	0.995
ln (Daily sugar from beverages)		139	3.5 (1.4)	310	3.5 (1.2)	0.627
Life less satisfying because of mouth/breath problems						
		Often/Sometimes		Rarely/Never		
Food before bed	No	75	30%	179	70%	0.796
	Yes	71	31%	161	69%	
Drink before bed	No	54	25%	159	75%	**0.046**
	Yes	92	34%	181	66%	
Food/drink before bed	No	43	27%	118	73%	0.259
	Yes	103	32%	222	68%	
ln (Daily sugar intake)		135	4.1 (1.1)	306	4.1 (1.1)	0.937
ln (Daily sugar from food)		135	2.9 (1.2)	306	3.0 (1.1)	0.714
ln (Daily sugar from beverages)		135	3.5 (1.3)	306	3.5 (1.2)	0.876
Sore or bleeding gums						
		Often/Sometimes		Rarely/Never		
Food before bed	No	80	31%	175	69%	0.053
	Yes	96	40%	146	60%	
Drink before bed	No	64	30%	150	70%	**0.026**
	Yes	112	40%	171	60%	
Food/drink before bed	No	45	28%	116	72%	**0.016**
	Yes	131	39%	205	61%	
ln (Daily sugar intake)		161	4.2 (1.2)	291	4.1 (1.0)	0.339
ln (Daily sugar from food)		161	2.9 (1.2)	291	3.0 (1.1)	0.464
ln (Daily sugar from beverages)		161	3.7 (1.3)	291	3.5 (1.2)	**0.036**
Prescription medication for oral or dental problems						
		No		Yes		
Food before bed	No	224	86%	35	14%	0.265
	Yes	209	83%	43	17%	
Drink before bed	No	191	88%	26	12%	0.076
	Yes	242	82%	52	18%	
Food/drink before bed	No	145	89%	18	11%	0.069
	Yes	288	83%	60	17%	
ln (Daily sugar intake)		396	4.0 (1.1)	70	4.5 (1.0)	**0.001**
ln (Daily sugar from food)		396	2.9 (1.1)	70	3.4 (1.1)	**0.003**
ln (Daily sugar from beverages)		396	3.5 (1.2)	70	3.9 (1.2)	**0.004**
Dental pain						
		No		Yes		
Food before bed	No	183	71%	74	29%	**0.028**
	Yes	155	62%	95	38%	
Drink before bed	No	155	72%	60	28%	**0.026**
	Yes	183	63%	109	37%	
Food/drink before bed	No	115	71%	46	29%	0.121
	Yes	223	64%	123	36%	
ln (Daily sugar intake)		302	4.0 (1.0)	162	4.3 (1.2)	**0.006**
ln (Daily sugar from food)		302	2.9 (1.1)	162	3.2 (1.2)	**0.018**
ln (Daily sugar from beverages)		302	3.4 (1.2)	162	3.8 (1.3)	**0.002**

**Table 4:** Multivariable analyses dental outcomes based on demographic characteristics and diet-related behaviors.

Diet-Related Behavior		Beta (SE)	*p*-Value	Odds Ratio (95% CI)
Condition of teeth and gums (Fair/Poor/Very Poor vs. Very Good/Good), AUC = 0.68 *				
Intercept		1.55 (0.45)	0.001	
Education level	increasing level	−0.25 (0.09)	0.005	0.8 (0.7–0.9)
Marital status	No vs. Yes	−0.18 (0.23)	0.420	0.8 (0.5–1.3)
Health insurance	Yes vs. No	−0.49 (0.28)	0.082	0.6 (0.4–1.1)
Dental insurance	Yes vs. No	−0.33 (0.29)	0.254	0.7 (0.4–1.3)
Food/drink before bed	Yes vs. No	0.78 (0.24)	**0.001**	**2.2 (1.4–3.5)**
ln (Daily sugar from beverages)		−0.02 (0.09)	0.805	1.0 (0.8–1.2)
Embarrassed because of mouth smell (Rarely/Never vs. Often/Sometimes), AUC = 0.63				
Intercept		0.03 (0.53)	0.949	
Education level	increasing level	−0.21 (0.09)	0.019	0.8 (0.7–1.0)
Marital status	No vs. Yes	−0.43 (0.23)	0.061	0.7 (0.4–1.0)
Health insurance	Yes vs. No	0.06 (0.27)	0.835	1.1 (0.6–1.8)
Dental insurance	Yes vs. No	−0.25 (0.28)	0.381	0.8 (0.4–1.4)
Food before bed	Yes vs. No	−0.50 (0.22)	**0.025**	**0.6 (0.4–0.9)**
ln (Daily sugar intake)		0.17 (0.11)	0.129	1.2 (1.0–1.5)
Sore or bleeding gums (Rarely/Never vs. Often/Sometimes), AUC = 0.62				
Intercept		−1.04 (0.45)	0.021	
Education level	increasing level	−0.08 (0.09)	0.342	0.9 (0.8–1.1)
Marital status	No vs. Yes	−0.19 (0.22)	0.393	0.8 (0.5–1.3)
Health insurance	Yes vs. No	−0.48 (0.28)	0.087	0.6 (0.4–1.1)
Dental insurance	Yes vs. No	0.33 (0.29)	0.255	1.4 (0.8–2.5)
Food/drink before bed	Yes vs. No	0.58 (0.25)	**0.019**	**1.8 (1.1–2.9)**
ln (Daily sugar from beverages)		0.13 (0.09)	0.142	1.1 (1.0–1.4)
Prescription medication for oral or dental problems (Yes vs. No), AUC = 0.63				
Intercept		−4.08 (0.83)	<0.001	
Education level	increasing level	−0.04 (0.12)	0.711	1.0 (0.8–1.2)
Marital status	No vs. Yes	0.00 (0.31)	0.997	1.0 (0.5–1.8)
Health insurance	Yes vs. No	0.40 (0.38)	0.284	1.5 (0.7–3.1)
Dental insurance	Yes vs. No	0.16 (0.38)	0.663	1.2 (0.6–2.5)
Drink before bed	Yes vs. No	0.32 (0.32)	0.328	1.4 (0.7–2.6)
ln (Daily sugar intake)		0.43 (0.16)	**0.008**	**1.5 (1.1–2.1)**
Dental pain (Yes vs. No), AUC = 0.63				
Intercept		−1.18 (0.45)	0.009	
Education level	increasing level	−0.20 (0.09)	0.023	0.8 (0.7–1.0)
Marital status	No vs. Yes	−0.33 (0.22)	0.138	0.7 (0.5–1.1)
Health insurance	Yes vs. No	0.04 (0.28)	0.875	1.0 (0.6–1.8)
Dental insurance	Yes vs. No	0.37 (0.28)	0.195	1.4 (0.8–2.5)
Drink before bed	Yes vs. No	0.23 (0.22)	0.298	1.3 (0.8–1.9)
ln (Daily sugar from beverages)		0.28 (0.09)	**0.003**	**1.3 (1.1–1.6)**

* *n* = 511; AUC = Area Under the Curve.
